# Addressing the gaps of the functional neuroimaging in young APOEe4 carriers: A systematic review and Graph Theoretical analysis of brain connectivity

**DOI:** 10.1002/alz.093652

**Published:** 2025-01-09

**Authors:** Ludmila Kucikova, Adam Brass, Xing Xiong, Jianmin Zeng, Craig Ritchie, Graciela Muniz‐Terrera, John T O'Brien, Li Su

**Affiliations:** ^1^ University of Sheffield, Sheffield United Kingdom; ^2^ Southwest University, Chongqing China; ^3^ Scottish Brain Sciences, Edinburgh United Kingdom; ^4^ Department of Social Medicine, Ohio University, Athens, OH USA; ^5^ University of Cambridge, Cambridge, Cambridgeshire United Kingdom

## Abstract

**Background:**

In the last decade, extensive research has emerged into understanding the impact of risk factors for Alzheimer’s Disease (AD) on brain function in pre‐symptomatic stages. Here, we focused on the apolipoprotein e4 (APOEe4) gene, the major genetic risk factor for sporadic AD, and its effect on brain function in early adulthood.

**Method:**

In the first part of the study, we systematically reviewed the multimodal functional neuroimaging literature, exploring its relationship with cognition, and the potential effects of other variables including the demographics, other risk factors, and methodological and analytical choices. While the studies demonstrated consistent alterations of APOEe4 carriers in brain connectivity and activity; the results of fMRI studies covered mostly the differences in the directionality using standard connectivity and activity measures. In the second part of this study, we aimed to address this gap by using the graph theory analysis to explore the dynamic behaviour of the six resting‐state networks of interest in young APOEe4 carriers versus non‐carriers (n = 129, aged 17‐22).

**Result:**

Average Path Length and Closeness Centrality were consistently disrupted, pointing to network reorganisation in multiple resting‐state networks, albeit using different mechanisms.

**Conclusion:**

This study is the first to demonstrate the restructuring of multiple resting‐state networks in young adults modulated by the APOE genotype.

